# Challenges of Rabies Surveillance in the Eastern Amazon: The Need of a One Health Approach to Predict Rabies Spillover

**DOI:** 10.3389/fpubh.2021.624574

**Published:** 2021-06-25

**Authors:** Victor Bastos, Roberta Mota, Mylenna Guimarães, Yuri Richard, André Luis Lima, Alexandre Casseb, Gyovanna Corrêa Barata, Jorge Andrade, Livia Medeiros Neves Casseb

**Affiliations:** ^1^Federal University of Pará, Institute of Biological Sciences, Belém, Brazil; ^2^Department of Arbovirology and Hemorrhagic Fevers, Evandro Chagas Institute, Ananindeua, Brazil; ^3^Federal Rural University of the Amazon, Institute of Animal Health and Production, Belém, Brazil; ^4^Amazon Metropolitan College, Belém, Brazil; ^5^Pará State Health Secretary, Health Surveillance Directorate, Belém, Brazil

**Keywords:** universality, equity, Amazon, human rabies, One Health

## Abstract

Brazil has been promoting essential improvements in health indicators by implementing free-access health programs, which successfully reduced the prevalence of neglected zoonosis in urban areas, such as rabies. Despite constant efforts from the authorities to monitor and control the disease, sylvatic rabies is a current issue in Amazon's communities. The inequalities among Amazon areas challenge the expansion of high-tech services and limit the implementation of active laboratory surveillance to effectively avoid outbreaks in human and non-human hosts, which also reproduces a panorama of vulnerability in risk communities. Because rabies is a preventable disease, the prevalence in the particular context of the Amazon area highlights the failure of surveillance strategies to predict spillovers and indicates the need to adapt the public policies to a “One Health” approach. Therefore, this work assesses the distribution of free care resources and facilities among Pará's regions in the oriental Amazon; and discusses the challenges of implanting One Health in the particular context of the territory. We indicate a much-needed strengthening of the sylvatic and urban surveillance networks to achieve the “Zero by 30” goal, which is inextricable from multilateral efforts to combat the progressive biome's degradation.

## Introduction

Brazil's seventh Constitution defined health services as a fundamental Brazilian right. This revolutionary and pioneering strategy, founded on social justice, sets up the basis for a public health system—Brazil's Unified Health System (SUS). The SUS was later regulated by law n° 8080/1990, which defines its principles: universality, equity, and integrity (1, 2). In summary, the system aims to guarantee free universal access to both essential care services and complex procedures such as surgeries to 140 million people throughout Brazil's territory (3). However, due to regional inequalities, ensuring those principles has been a challenge, especially in Amazon riverside communities that have been affected by neglected diseases (4).

The Brazilian Amazon composes about 49,3% of Brazil's area geographically and is distributed into nine states (5), which through law n° 291/1967 and law n° 356/1968, are divided into the Occidental and Oriental Amazon. The first division included four states, while the second division comprises five others, including Pará, in the country's northern region (6). These Amazonian states were developed unequally compared with Brazil's capitalist main poles, and its integration to International Trade started after the oil crises in 1973 initiated a disordered development process focused on limited economic spots (7) (p. 154) that strongly evoked social and territorial conflicts (8) (p. 46). This inequity is reflected by the low Human Development Index of all Amazonian states during the first decade of the 21st century (9), despite their international and national importance as a raw material provider.

Regarding the state of Pará, government strategies aiming to populate remote areas and develop its commercial activity during the 20th century created five different poles associated with mineral exploration, that are the center of Pará's Gross Domestic Product (7) (p. 157–159) (10) (p. 159). These economic strategies enhanced selective economic development, promoting regional, social, and economic inequities within different state areas related to mining and other activities (10, 11) (p. 158–164). It also started an intense human population growth, besides ecological transformation in the biome, which enhances human-wildlife interaction, and introduces communities to the cycle of wild pathogens, highlighting the importance of the One Health approach in the Amazon area (12–15).

One Health is a multisectoral and multidisciplinary approach that recognizes the close interaction between human and animal health (16). This approach is an essential tool for guiding the efforts of public policies in the prevention of zoonosis and has demonstrated efficient results toward the control of rabies in endemic areas (17). As such, there is a need for close and continuous vigilance of at-risk populations that demand cross-sector cooperation, including proactive surveillance of animal vectors such as dogs and bats, which play an essential role in the transmission of rabies.

Rabies is an acute infectious encephalitis caused by a neurotropic virus from the *Lyssavirus* genus, which can infect all mammalian hosts, leading to death in almost all cases. Its transmission to humans occurs mainly through a bite from an infected domestic or wild host (18, 19). Despite its preventable aspects, rabies threatens almost 60,000 humans globally, especially in Africa and Asia (20). In Brazil, the Rabies Prophylaxis Program (PNPR) achieved actual progress in the 21st century toward controlling urban rabies through post-exposure prophylaxis (PEP) schemes. However, rabies transmitted by bats is a current issue: from 2003 to 2018, 143 fatal cases mainly transmitted by wild vectors were reported. There was a high level of transmission in the state of Pará, the second highest endemic area in the country (4, 21–23). The groups most affected by rabies in the Amazon were those living in neglected zones, where equitable public health services are not available, emphasizing this illness's neglected profile (4, 20).

The occurrence of human rabies cases in neglected communities suggests the failure of surveillance strategies, and it indicates the need for improvements in the parameters of the public health system to achieve WHO's “zero by 30” goal. Therefore, the work aims to assess: (i) the distribution of health services from different levels in the context of rabies prevention and (ii) the challenges of implementing a “One Health” approach in the Amazon.

## Materials and Methods

### Data Collection

A descriptive, observational, and cross-sectional research was carried out to assess data on the sufficiency of free essential care resources available to assist risk communities in Pará zones from 2018 to 2019. We collated (i) the distribution of health units based on their complexity levels; (ii) surveillance data on domestic and wildlife animal-bite reported during the period, (iii) availability of human rabies vaccine; and (iv) the distribution of health centers for diagnosis. Data about both health units and the availability of rabies vaccines are public-access and can be formally requested from the State Health Secretary of Pará (SESPA) by every health professional through the institutional e-mail (protocolo@sespa.pa.gov.b). The request was processed as PAE 2020/434223 and accepted on June 25, 2020. We obtained surveillance data on animal bites from the “Individual Investigation Reports of Human anti-Rabies Care” form filled by health workers at health units and submitted to SINAN (24).

This document must be completed by nurses and other health professionals each time a patient seeks care after animal aggression, and it is sent weekly from municipalities to state levels and biweekly to the Health Ministry (MS). They are obligated to investigate and finalize cases within 2 months. The investigations may follow PEP administration depending on the type of injury and monitoring the potential rabid dog; upon direct contact with a sylvatic animal, the patient receives five doses of intradermal rabies vaccine (cell culture) on days 0, 3, 7, 14, and 28 (25). It is concluded when the patient interrupts the treatment or when PEP finishes.

This work assessed the total applied doses of only human rabies vaccines (cell culture/Vero and cell culture/embryo) and the animal bite reports on SINAN in the whole state from 2018 to 2019. The data unavailability on the type of doses (1st dose, 2nd, 3rd, four, or booster), and the absence of the profile of animal bite reported from the municipalities limits the article's conclusions.

**Figure 1:**
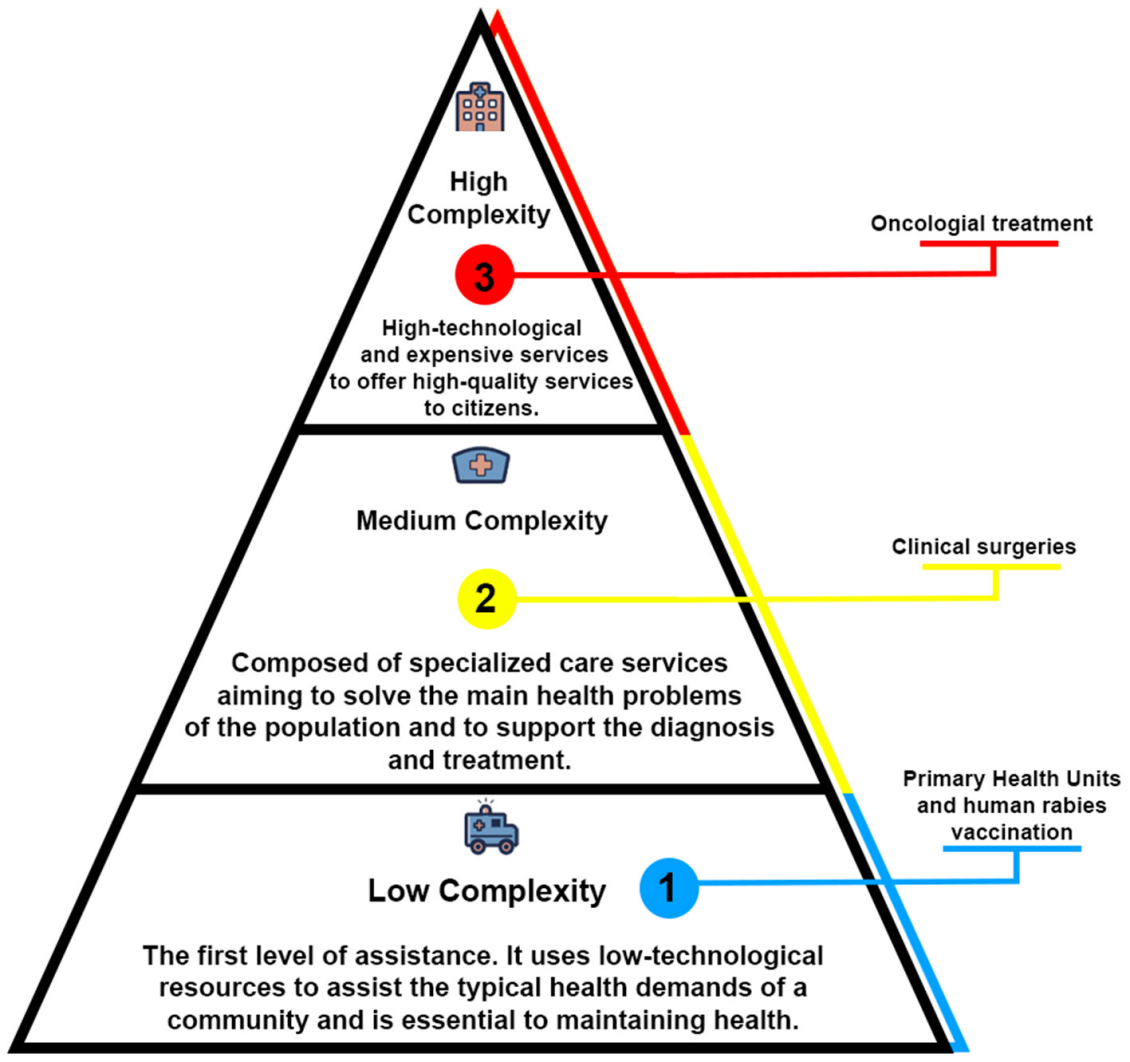
The levels of health care in the context of Brazil's system. The organizational health system's model follows the WHO's recommendations to divide levels of assistance according to health units' resources available to meet the patient's demands. The primary level works in a preventable approach by promoting close contact with the community. It may also offer free access to vaccines and medicine. The second level comprises hospitals and care centers that provide ambulatorial assistance to solve the main health problems. The third level offers high-cost and high-specialized treatments for the patient's rehabilitation, including Intensive Care Units and oncologic treatment.

In order to analyze the distribution of free care resources among the different zones in Pará, we compiled health unit information based on their level (Figure 1), following the Primary attention, Second and Third attention (26). This division is based on health services' organizational arrangements from different technological levels, which through integrated collaboration, seek to guarantee free care services.

### Study Area

This study covered the state of Pará, located in the north of Brazil, to the Oriental Amazon (6). The state area is about 1,245,870,707 km^2^ and has an estimated population of 8,690,745 people (5).

**Figure 2:**
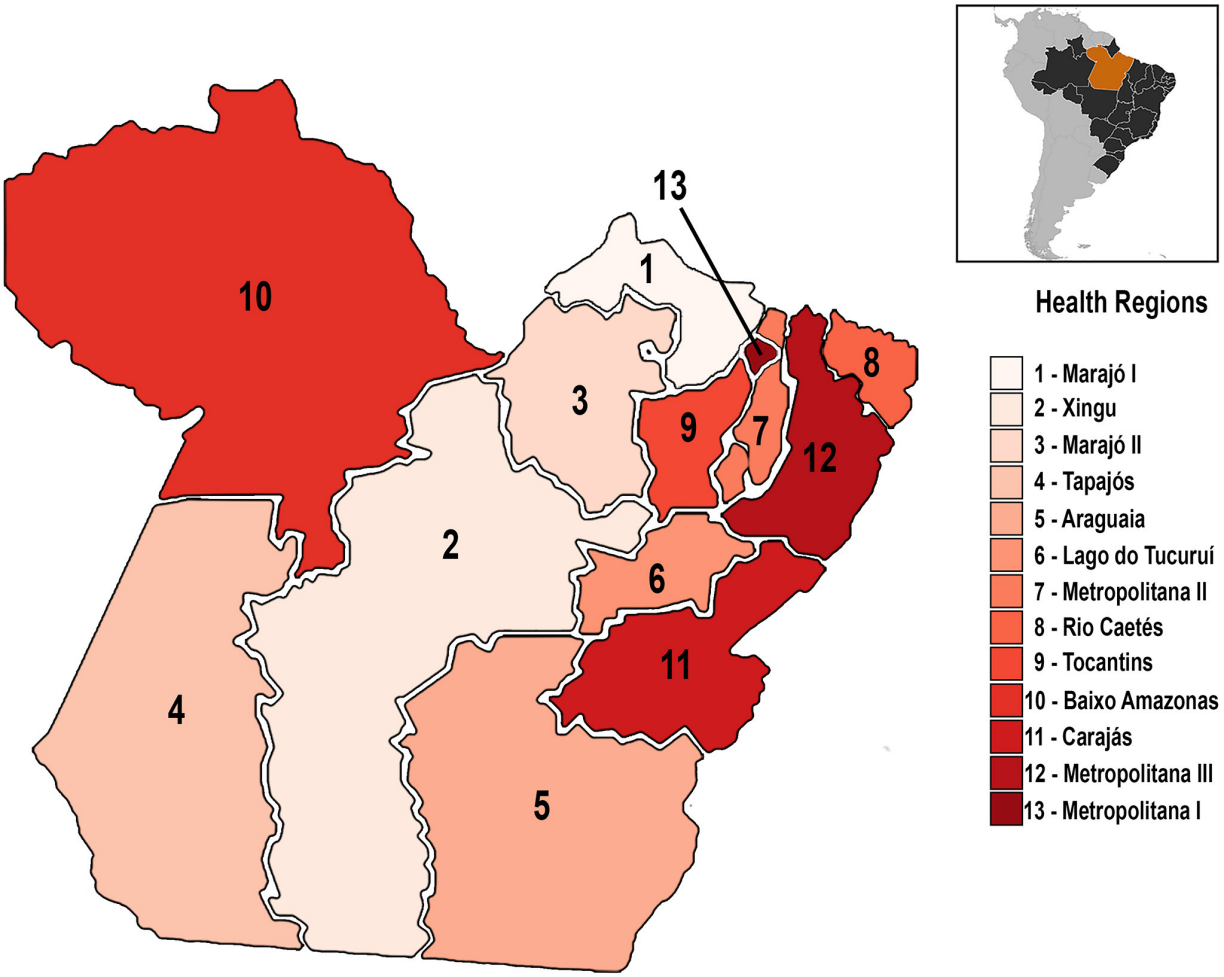
Pará's administrative division (SESPA). The regionalization of care resources is a national effort to reduce health inequities among Brazil's regions. In the Pará state, there are 13 health regions, which comprise the 144 municipalities of the area, considering their geographical characteristics.

The analysis followed data available from the municipalities arranged in the 13 Health Regions, which SESPA defined according to the Resolution CIB/PA n° 90, from June 12, 2013: Araguaia, Baixo Amazonas, Carajás, Lago de Tucuruí, Marajó I, Marajó II, Metropolitana I, Metropolitana II, Metropolitana III, Rio Caetés, Tapajós, Tocantins e Xingú, covering 144 municipalities (Figure 2).

This administrative division aims to improve the distribution of free medical resources among the municipalities and provide equal access to free care services. The division also considers the neighboring areas' geographical and demographic aspects to include them in the same health region.

### Epidemiological Data

The data on human rabies epidemiological status in Brazil is open access and is available on the Datasus tabnet platform (http://www2.datasus.gov.br/DATASUS/index.php). This database collects, organizes, and offers Brazil's health information, including epidemiological data on infectious diseases; in which human rabies is inserted.

### Statistical Analysis

We performed a descriptive analysis of the distribution of primary care units, rabies vaccines, and medium and high-level hospitals by calculating the differences in the rate per 10^4^ people or 10^5^ inhabitants. The regional rate of medical services was determined by the ratio of total health units and regional population per 10,000 or 100,000 inhabitants.

(1) $$\begin{array}{l} rate=\left(\frac{total\;of\;health\;units}{regional\;population}\right)x\;10,000\;or\;100,000 \end{array}$$

Statistical analysis was performed with Graph Pad Prism 8th version for Windows 10. The mean human rabies' offer compared to the total of animal-bite notification by Wilcoxon Signed Rank Test to assess the availability of free medical resources among the regions. We performed a One-way ANOVA to analyze the efficiency of the PEP scheme by comparing the mean of concluded and non-concluded treatments with the total notifications.

The differences were considered significant at a 95% confidence interval (*p* < 0.05).

### Populational Data

Data on the Brazilian states' and municipalities' population is public access information, and it is available on the website of IBGE (https://cidades.ibge.gov.br/). The complete sociodemographic information of every Brazilian area can be accessed by searching the state and the cities' names on the website. To perform the analysis on the distribution of health services according to their levels per 10^4^ or 10^5^ inhabitants, we add each municipalities' population to estimate the total inhabitants of each health region.

## Results

### The Distribution of Primary Care Units Among the Health Regions of Pará State

According to Brazil's Health Ministry's recommendation, the primary health units should assist an area of 12–18 thousand inhabitants in big cities, depending on the services offered to the community (Ministério da Saúde). Thus, when we assessed the distribution among Pará's area, we observed a homogenous distribution of these units among the state's areas, varying from 1 primary health unit/10^4^ inhabitants to 4 primary health units/10^4^ inhabitants (Table 1). The riverine area, Marajó I (4,3/10,000), has the most significant distribution of primary health care units, followed by Tapajós (4,3/10,000) and Rio Caetés (4,2/10,000). In contrast, the Metropolitana I region (0.9/10,000), which concentrates the biggest population among the areas, had the lowest distribution, almost three times lesser than Marajó I and Tapajós.

**Table 1:** The distribution of primary health units among Pará's regions.

**Health regions (SESPA)**	**Total of primary health units**	**Distribution/10^**4**^ inhabitants**	**Regional population (2019)**
Araguaia	194	3.42/10^4^ people	566,682
Baixo Amazonas	252	3.26/10^4^ people	771,715
Carajás	204	2.33/10^4^ people	875,232
Lago de Tucuruí	116	2.51/10^4^ people	461,593
Metropolitana I	205	0.91/10^4^ people	2,238,680
Metropolitana II	142	3.86/10^4^ people	367,592
Metropolitana III	375	3.99/10^4^ people	939,421
Rio Caetés	231	4.26/10^4^ people	541,251
Tapajós	97	4.38/10^4^ people	221,135
Tocantins	193	2.73/10^4^ people	705,089
Xingú	134	3.82/10^4^ people	350,276
Marajó I	105	4.30/10^4^ people	244,027
Marajó II	115	3.59/10^4^ people	320,172

*The analysis on the mean coverage of services from the Primary Health Care (PHC) indicates a homogeneous distribution among the areas. It considered data on the availability of Family Health Support Center (CASF), primary health units, health center, home care, indigenous health care units, and fluvial mobile units*.

### Differential Availability of Specialized Medical Resources and the Concentration of High-Tech Resources in Urban Centers

**Figure 3:**
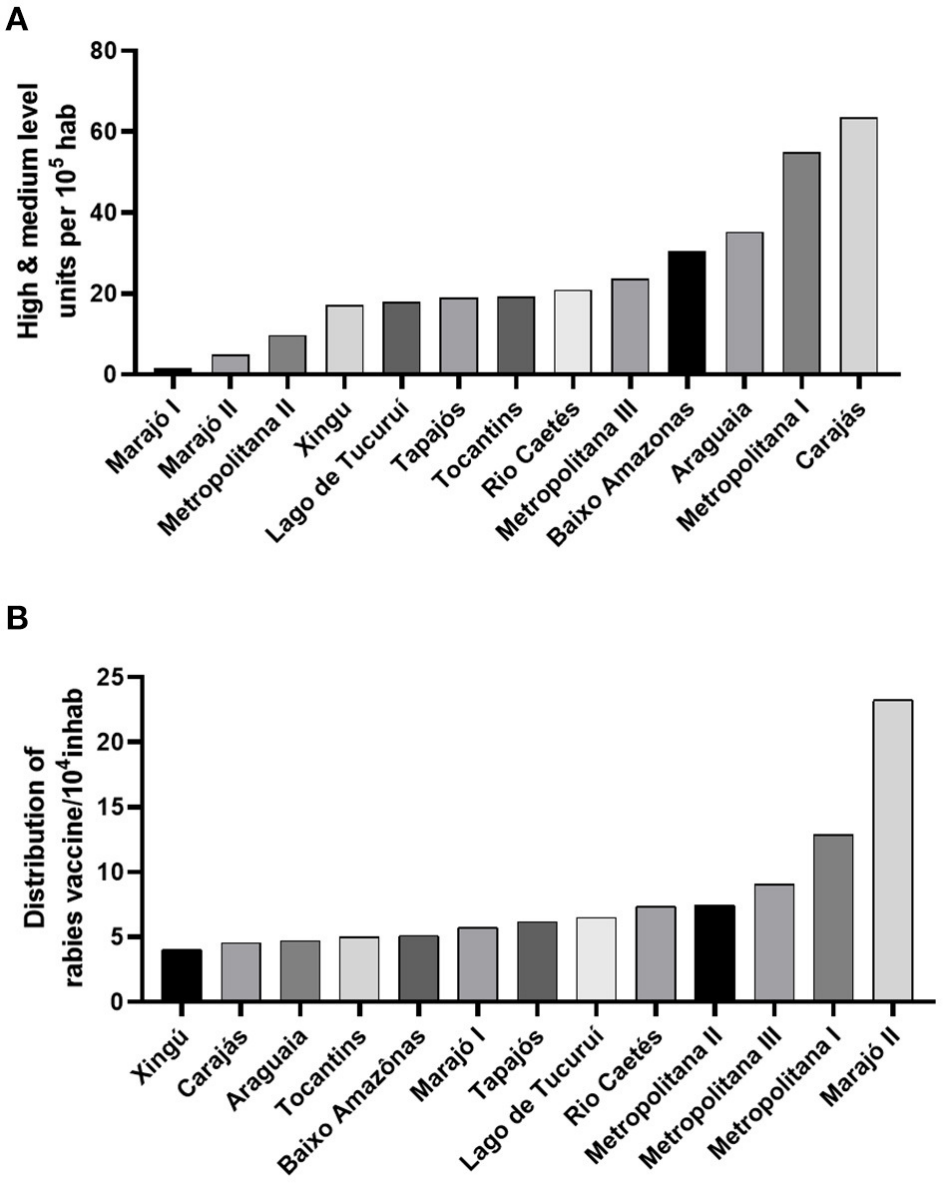
The availability of free medical resources and facilities. **(A)** human rabies vaccine's distribution per 10,000 people is heterogeneous and limited in some areas of the state. **(B)** the coverage of hospitals from medium and high-technological resources is concentrated in a few areas of the territory, and almost absent in neglected communities.

The availability of specialized care services provided by hospitals and emergencies from medium and high technological-level greatly varied per 100,000 people among the health regions (Figure 3A). Neglected areas, such as Marajó I (1,6/100,000) and Marajó II (5/100,000), had the worst indicators, followed by Metropolitana II (9,7/100,000). The greatest indicators were concentrated in a few urban areas, such as Carajás (63/100,000) and Metropolitana I (55/100,000)—which respectively concentrate 39 and 34 times more hospitals than Marajó I.

Among the health regions, the Metropolitana I region concentrates the only laboratory which supports animal and human rabies antemortem and postmortem diagnosis in the Amazonia, the Instituto Evandro Chagas, located at Ananindeua city (1° 21′ 59″ S, 48° 22′ 20″ W). Besides supporting Pará's demand, the laboratory has a central role in supporting rabies surveillance in the North area of the country.

### The Offer of Cell Culture Human Rabies Vaccines

Rabies prophylaxis may be administered in two primary schemes: Post-Exposure Prophylaxis (PEP) and Pre-Exposure Prophylaxis (PrEP) by applying intradermal doses of human rabies vaccine raised in cell culture (27). Among Pará's regions, the availability of this resource in the health units varied from 4 vaccines/10,000 people—such as in the Araguaia (4,69/10,000), Carajás (4,55/10,000) and Xingú (4,02/10,000) -, to 23/10,000 people in the Marajó II areas (Figure 3B).

**Figure 4:**
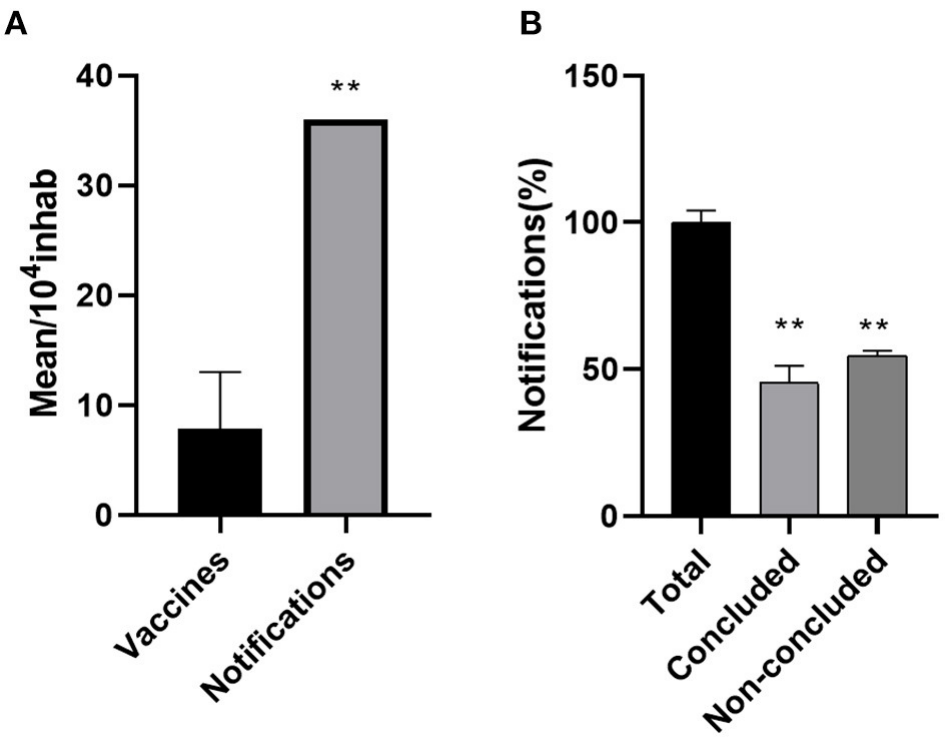
Reports on animal bite and PEP administration in the Pará state during 2018 and 2019. **(A)** the offer of rabies vaccines may be insufficient to assist the PEP demand in some neglected areas of the state ^**^(*p* < 0.01). **(B)** the reports on patients seeking care after a sylvatic or domestic animal bite indicate the high rate of interruption in the PEP ^**^(*p* < 0.01).

The total average of the doses available in the regions was compared with the total average of animal-bite notifications in SINAN. Our data indicate that the availability of human rabies vaccines may be insufficient to assist the local demands (Figure 4A).

### Reports on Animal-Bite Assistance and PEP Administering in the Pará Areas

In 2018, there were 33, 549 cases of wild and domestic animal bite-notifications reported in the SINAN database, of which 17,029 patients (50,7%) did not finish the treatment without interruptions. In 2019, there were 30,970 notifications of animal injury, in which 18,163 (58%) did not follow the complete PEP scheme. The differences between concluded and non-concluded PEP were both considered significant (*p* < 0.05) to the total average of notifications (Figure 4B).

### The Epidemiological Profile of Rabies in the Amazonia: Central Role of the State of Pará in Human Rabies Epidemiology

**Figure 5:**
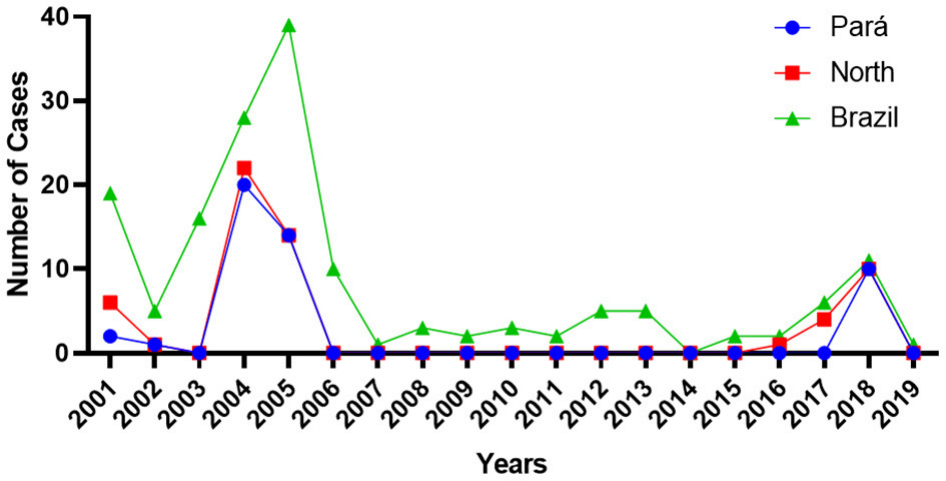
Human rabies epidemiology in northern Brazil (2000–2018). It was reported 160 fatal cases in the period, with bat-transmitted outbreaks. Pará state has a central role in the northern region, representing 81% of the total confirmed cases.

In Brazil, 160 fatal cases of human rabies were reported from 2001 to 2018, in which 58 cases (36%) have occurred in the North (Figure 5). Most of the cases that occurred in the northern region were reported in the state of Pará, which totaled 47 cases (81%) of rabies, occurred in 2001 and 2002, in the Carajás region (three cases); in 2004, in the Metropolitana I (two cases), Rio Caetés (three cases), and Marajó II (15 cases). In 2005, 14 cases were reported among Metropolitana I (eight cases) and Rio Caetés (six cases). More recently, a bat-transmitted rabies outbreak was reported in the Marajó II region, which confirmed ten fatal cases.

## Discussion

Brazil has achieved improvements in health indicators by implementing and expanding free healthcare programs, in which Primary Health Care (PHC) are the protagonists (28). PHC, mainly represented by primary health units, considers the socio-cultural aspects of the area coverage to promote community and family orientation by health educational strategies in a close-contact approach and plays an essential role in promoting health, especially in the pandemic's context (29). Similarly, some health programs, such as the national rabies prophylaxis program, have contributed significantly to reducing human mortality through dog and cat vaccination campaigns, besides implementing pre-exposure prophylaxis (PrEP) and post-exposure prophylaxis (PEP) strategies. Despite Brazil's efforts toward controlling urban rabies and achieving “Zero by 30” WHO's goal, rabies transmitted by wild vectors, such as bats, are also a current issue in the Amazon areas (30). Rabies reemergence in neglected communities has a close relation with anthropic destruction of animals' habitats and health, affecting disease transmission dynamics (13, 15, 31, 32). The occurrence of a bat-transmitted human rabies outbreak in 2018 at Melgaço's riverside areas and throughout other Amazon areas suggests a failure of rabies surveillance and health systems to deal with rabies considering the availability of preventable strategies to avoid the occurrence in animals and humans.

Therefore, this work assessed the distribution of free care services and resources among Pará's health regions. Health and socioeconomic inequities are challenges to achieving the universality of health policies and are aggravated by the limited governance of health authorities in neglected areas, particularly in the Northern region (33). Health services' regionalization may serve as an alternative to reduce inequities in free care services access (34). Unlike Andrade et al. (14), and Garnelo et al. (35) that discuss the limited PHC's distribution in the Amazon areas, in the particular context of the state of Pará, we demonstrated a homogeneous distribution of primary health units and primary health care centers among the regions of Pará. However, this data may not reflect the reality of these municipalities since we could not assess this distribution within their context, but at the regional level, limiting the analysis of particular distribution in remote areas. Although there were no discrepancies in the coverage of health units among the areas of Pará, other variables, such as the distribution of health professionals in urban and rural areas, may influence the quality and offer of essential care services, impacting the correct guidance treatment of aggravations in remote zones. That is because the northern region, including the capitals, has the worst indicators of distribution of doctors per thousand inhabitants among Brazil's regions (36). This indicates a panorama of greater vulnerability, with difficulties in access to physicians. However, health inequalities cannot be analyzed only by focusing on the PHC but also by considering other levels (medium and high) of health assistance (37).

Moreover, the limitation of the high-technological service in the particular Amazon scenario is a historical issue. The progress in reducing poverty and inequality in the 2000s had a paradoxical effect on Brazilian territory since the developmental agenda focused on activities related to the geographical specificities of the macro-regions contributing to spatial inequalities (38). This strategy enhanced the social conflicts in the area (8), and reproduced the same pattern in the mesoregions of Pará (7). Initially, it also affected the distribution of health services in remote zones: health services from the medium and high level remained concentrated in few developed areas (39), differently from the distribution of primary health units—that was significantly expanded in the poorest regions of the country, with greater limitations for its implementation in densest metropolitan peripheries, similarly to our results (Table 1). Despite Brazil's efforts to reduce vulnerability in recent decades, current data still demonstrate a concentration of these specialized health services (40). There is a considerable difference in the mean-coverage of hospitals of medium and high-level complexity among rural and urban areas of the Pará: the availability of specialized medical resources is almost 35 times greater in Metropolitana I (54.9/100,000) than Marajó I (1.6/100,000), and 11 times greater than Marajó II (4.9/100,000), affecting the access of neglected communities to specialized care resources. The unequal distribution of medical resources indicates the need for a large displacement between the regions to seek medical assistance, which directly influences the time for receiving adequate treatment in case of accidents; and compromises the maintenance of a patient's life. However, from other urban areas, riverside communities have particular barriers in transporting to the nearest hospital since it may be influenced by the hydrological cycles of drought and flooding, besides climatic conditions (35). Based on this scenario, in 2017, Brazilian authorities implemented the Fluvial mobile units (FMU) aiming to guarantee integrated health assistance to these communities (41). However, it could not meet more complex demands such as hospitalizations, the treatment, and diagnosis of infectious diseases, such as human rabies.

Regarding rabies diagnosis, the WHO indicates that the laboratory should be involved beyond human and animal diagnostic, maintaining a proactive role in the investigation, planning, and assessment of rabies cases using a One Health approach (42). Successful strategies in Latin America toward rabies control are based on dog-maintained RABV monitoring molecular sequencing, phylogenetic analysis, and antigenic typing to provide information on viral variants or lineages linked with its reservoir hosts—that is useful to mapping risk areas and help to implement surveillance strategies (43). The governments must also provide funding to implement decentralized networks for rabies surveillance and prevention, responsible for collecting samples to submit to specialized laboratories on the diagnosis, and the laboratories should work in a decentralized-network manner to support wild and human monitoring to expedite local strategies toward control and prevention (44).

For example, In Nepal, a coordinated approach implemented a laboratory network with five regional laboratories located in critical areas. It has been successfully typing and identifying the disease's epidemiological profile, which guides the implementation of preventable strategies. However, in the context of the Amazon, the limited distribution of laboratories working on animals' support (postmortem) and humans (ante- and postmortem) diagnosis represents a challenge to both rabies monitoring in the region and human rabies management, since a patient with rabies requires a complex structure, with an intensive care unit, and constant laboratory monitoring (45). The centralized laboratory networking may widely affect rabies surveillance by impacting the time in which results can be delivered and consequently delaying the health system's response to the risk of exposing naive hosts to the virus. The identification of positive cases needs a quick data reporting scheme for rapid decision-making (46), and a rapid animal's diagnosis with phylogenetic analysis may also affect the need for human post-exposure prophylaxis administration since the effective identification of risk zones can guide the implementation of preventable strategies, such as PrEP administration.

It would be interesting that emerging-endemic neighboring countries in Latin America, such as Brazil, Peru, and Bolivia, outline multilateral efforts to finance new epidemiological monitoring networks. It should also include sharing technological tools for the diagnosis, prevention, genetic, and serological typing to identify, guide, and perform strategies directed at areas of greater risk of spillover, especially in Amazon neglected zones. This international networking is already established in Europe (47), by the Middle East and Eastern Europe Rabies Expert Bureau (MEEREB), and in the Northern hemisphere, by Canada, United States, and Mexico through the North American Rabies Management Plan (NARMP) (48), which successfully achieved the control in endemic zones (49). It is noteworthy that monitoring rabies in the Amazon has beyond social but also economic importance since bovine livestock production has substantially increased throughout the decades, and the increase of cattle communities is related to the greater risk of human and animal exposure to RABV (50), especially in zones related to extensive deforested areas, large herds of cattle, and the presence of highways (51).

Similarly, the availability of essential resources, such as human rabies vaccines (Figure 4A), may not be satisfactory to meet the populational demands in some areas, especially in Carajás, Xingú, and Araguaia, which had the lowest distribution among the other regions (Figure 4B). Under the PEP plan, an animal bite may require an intradermal administration of cell-culture vaccines (27), and the patient must immediately seek medical assistance at a primary health unit or emergency center for receiving the correct PEP. Depending on the severity of the injury or the animal's aggressive characteristics (52), the treatment may be followed by RIG's administration (53). However, the long-term aspect of rabies prophylaxis, which involves multiple vaccine administrations at different times (27), and the low availability or the centralization of essential resources in some health units, can affect the efficiency of PEP. In addition to health inequities, geographic, cultural, and social aspects must be considered and may reflect low treatment continuity. For example, the insufficiency of knowledge about rabies in remote Amazon areas may play an important role in seeking care after an animal bite injury (54). Together, these aspects might explain the differences shown in Figure 5, which indicate that only half of the patients concluded the vaccination plan Nevertheless, the challenges of correctly following the PEP and guaranteeing essential resources are not a restricted issue in just the Amazonian reality but also a general context. Appropriate PEP use was also limited in China (55), India (56), and in other countries of Asia and Africa, in which the high cost and limited availability of the vaccine are the main barriers to receiving the correct PEP (57).

It highlights the importance of international efforts to reallocate resources to produce and distribute essential health supplies to vulnerable areas since well-succeeded countries have widespread access to rabies vaccines and control of rabies (57). Administering PrEP in at-risk communities in Latin America must be considered since it is an efficient strategy adopted in Peru and other countries (58). It has a cost-effective aspect, which may reduce the need for PEP with vaccination schemes. These efforts must be accompanied by extensive animal vaccination campaigns, including livestock vaccination, with the monitoring of animal herd immunity, followed by the control of cat and dog populations. Simple initiatives, such as promoting ample health education campaigns, may effectively reduce non-conformities on PEP administration and help expand the populational adherence to the animal's campaigns.

Endemic and emerging countries should also be proactive in mapping and monitoring health inequities (59) to implement public policies in at-risk areas. In the particular context of the neglected Amazonian areas, health policies need to embrace resource distribution and promote access to health services opportunities (60, 61), considering the socio-cultural heterogeneity and the geographic aspect of the territory (62). The heterogeneous distribution of high-tech biomedical resources among these highly diverse zones and the limited laboratory network denotes a barrier to the implementation of a proactive “One Health” approach in the Amazonian context since it requires constant animal, human, and vector surveillance (17), which become complex with an insufficient laboratory network. This active monitoring has been efficient in predicting and detecting the circulation of RABLV strains in the Ceará state (24), and the surveillance and control of sylvatic rabies is a crucial strategy in North America (63). These barriers may be overcome in the long-term if strategies that involve multilateral efforts between the different sectors of the government, states, and municipalities strengthen and establish new decentralized monitoring networks in different areas of the Brazilian territory. This can contribute to a positive outcome not only in the context of rabies but also in the prediction of emerging and reemerging infectious diseases in the Brazilian Amazon (12).

Therefore, this paper indicates a must-needed improvement in the health indicators and surveillance strategies in rabies reemergence, mainly in the particular scenario of the Amazon, since there are inequities in access to rabies treatment and vaccines in the neglected areas of the state. These rabies-based inequities are due to both poor access to health services in these communities, and the environmental exploitation that present government policies have which increased the contact of naïve hosts to wild vectors. Thus, to control human rabies in endemic areas and help achieve the “zero by 30” WHO goal, it is essential that Brazil's government promotes equitable policies and play a proactive role in monitoring RABLV circulation. Therefore, these efforts also require a constant commitment by public entities to protect the Amazon biome in its entirety, which is inextricable of animal's and human health.

## Data Availability Statement

The original contributions generated for this study are included in the article/supplementary material, further inquiries can be directed to the corresponding author/s.

## Author Contributions

VB, AC, and LC idealized and designed the research. VB draft the paper. VB, AL, and YR made the images. VB, MG, YR, AL, RM, GB, and JA analyzed and processed the data. VB and YR performed the statistics. VB, RM, and AL edited the paper. All the authors have reviewed and accepted the submitted manuscript version.

## Conflict of Interest

The authors declare that the research was conducted in the absence of any commercial or financial relationships that could be construed as a potential conflict of interest.
